# The future of vitamin D analogs

**DOI:** 10.3389/fphys.2014.00122

**Published:** 2014-04-03

**Authors:** Carlien Leyssens, Lieve Verlinden, Annemieke Verstuyf

**Affiliations:** Clinical and Experimental Endocrinology, Department of Clinical and Experimental Medicine, KU LeuvenLeuven, Belgium

**Keywords:** vitamin D, analogs, pleiotropic effects, cancer

## Abstract

The active form of vitamin D_3_, 1,25-dihydroxyvitamin D_3_, is a major regulator of bone and calcium homeostasis. In addition, this hormone also inhibits the proliferation and stimulates the differentiation of normal as well as malignant cells. Supraphysiological doses of 1,25-dihydroxyvitamin D_3_ are required to reduce cancer cell proliferation. However, these doses will lead *in vivo* to calcemic side effects such as hypercalcemia and hypercalciuria. During the last 25 years, many structural analogs of 1,25-dihydroxyvitamin D_3_ have been synthesized by the introduction of chemical modifications in the A-ring, central CD-ring region or side chain of 1,25-dihydroxyvitamin D_3_ in the hope to find molecules with a clear dissociation between the beneficial antiproliferative effects and adverse calcemic side effects. One example of such an analog with a good dissociation ratio is calcipotriol (Daivonex®), which is clinically used to treat the hyperproliferative skin disease psoriasis. Other vitamin D analogs were clinically approved for the treatment of osteoporosis or secondary hyperparathyroidism. No vitamin D analog is currently used in the clinic for the treatment of cancer although several analogs have been shown to be potent drugs in animal models of cancer. Transcriptomics studies as well as *in vitro* cell biological experiments unraveled basic mechanisms involved in the antineoplastic effects of vitamin D and its analogs. 1,25-dihydroxyvitamin D_3_ and analogs act in a cell type- and tissue-specific manner. Moreover, a blockade in the transition of the G0/1 toward S phase of the cell cycle, induction of apoptosis, inhibition of migration and invasion of tumor cells together with effects on angiogenesis and inflammation have been implicated in the pleiotropic effects of 1,25-dihydroxyvitamin D_3_ and its analogs. In this review we will give an overview of the action of vitamin D analogs in tumor cells and look forward how these compounds could be introduced in the clinical practice.

## Introduction

The active form of vitamin D_3_, 1,25-dihydroxyvitamin D_3_ **[**1α,25(OH)_2_D_3_; **1]** (Table 1), is mostly known for its effects on bone, calcium, and phosphate homeostasis. Next to these classical effects, 1,25(OH)_2_D_3_ also exerts so-called non-classical effects on various tissues which express the vitamin D receptor (VDR) as well as the enzymes that are responsible for activating the hydroxylations of vitamin D_3_, which is essential for the formation of 1,25(OH)_2_D_3_. Thus, most tissues have the ability to convert vitamin D_3_ into its active form, 1,25(OH)_2_D_3_, which in turn will bind the VDR in order to positively or negatively influence target genes via binding of the 1,25(OH)_2_D_3_/VDR complex to vitamin D receptor elements (VDRE). Non-classical properties of 1,25(OH)_2_D_3_ include prodifferentiating and antiproliferative effects on normal and cancer cells (Colston et al., 1981; Jensen et al., 2001) as well as immunomodulatory effects. However, in order to obtain these non-classical effects, 1,25(OH)_2_D_3_ doses of the nanomolar range are necessary, while physiological 1,25(OH)_2_D_3_ serum concentrations are in the picomolar range. Since supraphysiological doses of 1,25(OH)_2_D_3_ result in hypercalcemia, 1,25(OH)_2_D_3_ analogs were developed to minimize the calcemic side effects while preserving or augmenting the beneficial effects of 1,25(OH)_2_D_3_. Both industry and academic institutions have synthesized a vast amount of vitamin D analogs. Some of these analogs have tissue-specific effects with low calcemic side effects and can be given at higher doses compared to the mother compound.

**Table 1:** **Overview of vitamin D analogs**.

**Identification number**	**Name**	**Structure**
**[1]**	1α,25(OH)_2_D_3_	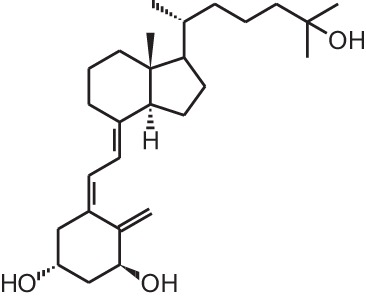
**[2]**	Paricalcitol (19-nor-1α,25(OH)_2_D_2_)	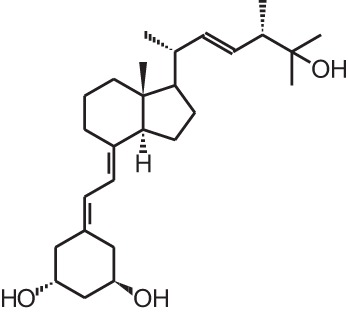
**[3]**	Doxercalciferol (1α(OH)D_2_)	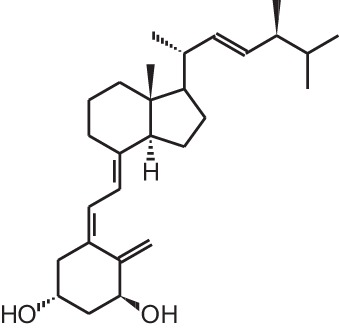
**[4]**	Falecalcitriol (26,27 F6-1α,25(OH)_2_D_3_)	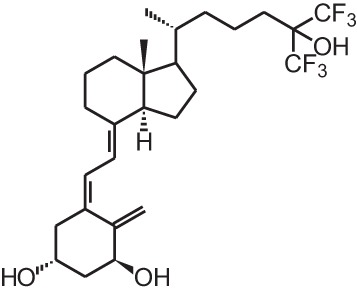
**[5]**	Maxacalcitol (22oxa-1α,25(OH)_2_D_3_)	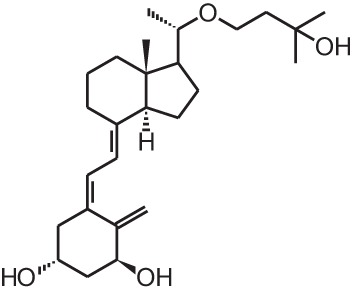
**[6]**	Tacalcitol (1α,24(R)(OH)_2_D_3_)	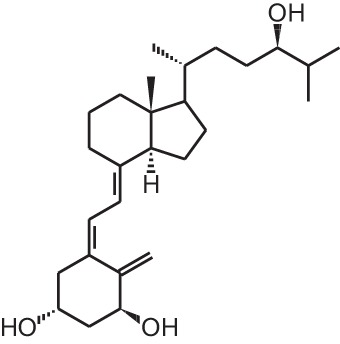
**[7]**	Calcipotriol (22-ene-26,27-dehydro-1α,25(OH)_2_D_3_)	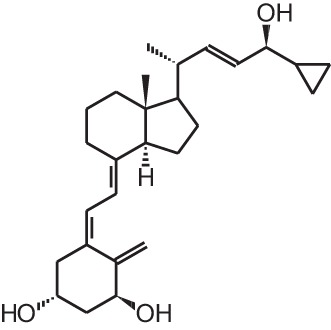
**[8]**	Alfacalcidol (1α(OH)D_3_)	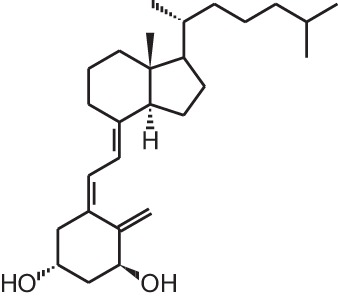
**[9]**	Eldecalcidol (2β-(3-hydroxypropoxy)-1α,25(OH)_2_D_3_)	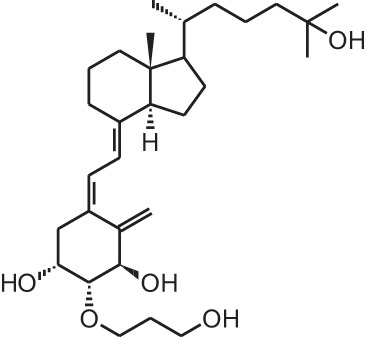
**[10]**	Seocalcitol (22,24-diene-24,26,27-trishomo-1α,25(OH)_2_D_3_**)**	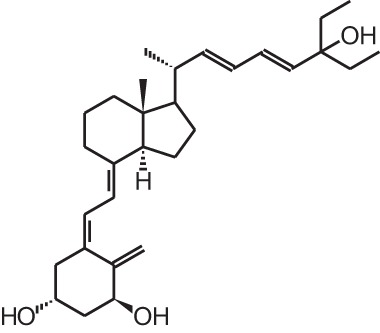
**[11]**	20-epi-1α,25(OH)_2_D_3_	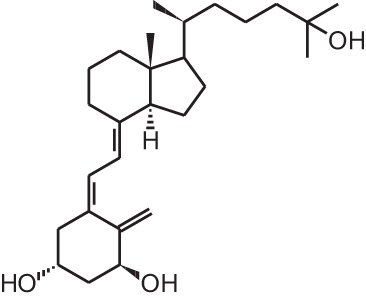
**[12]**	Lexicalcitol (20-epi-22-oxa-24,26,27-trishomo-1α,25(OH)_2_D_3_)	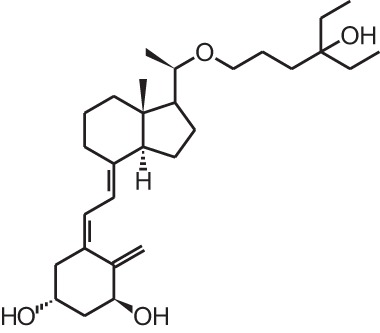
**[13]**	CD578 (17-methyl-19-nor-21-nor-23-yne-26,27-F6-1α,25(OH)_2_D_3_)	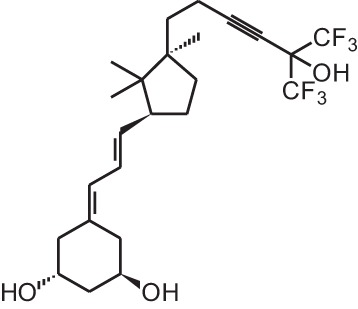
**[14]**	Inecalcitol (19-nor-14-epi-23-yne-1α,25(OH)_2_D_3_)	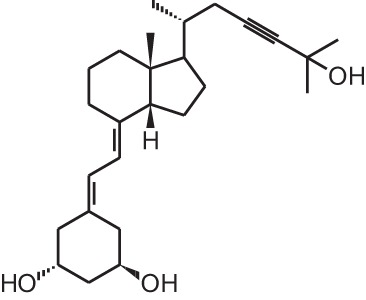
**[15]**	TX527 (19-nor-14,20-bisepi-23-yne-1α,25(OH)_2_D_3_)	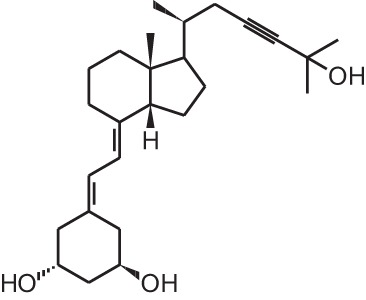
**[16]**	2MD (2-methylene-19-nor-(20S)-1α,25(OH)_2_D_3_)	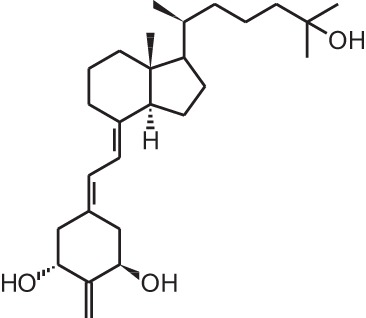
**[17]**	WY1112 (Seco-C-9,11-bisnor-17-methyl-20-epi-26,27-F6-1α,25(OH)_2_D_3_)	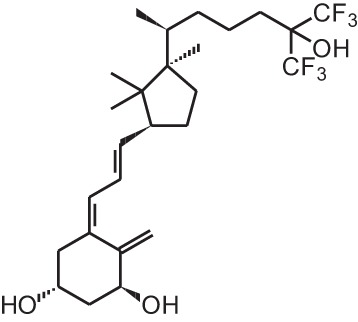
**[18]**	PRI-2205 ((5E,7E)-22-ene-26,27-dehydro-1α,25(OH)_2_D_3_)	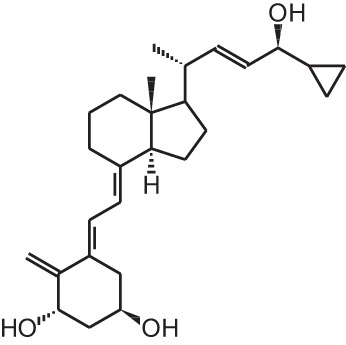
**[19]**	ILX23-7553 (16-ene-23-yne-1α,25(OH)_2_D_3_)	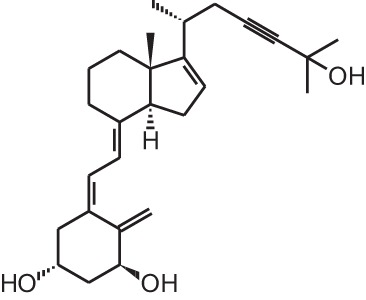

## Clinically approved vitamin D analogs

Given the huge amount of vitamin D analogs that have been synthesized during the years, it is nearly impossible to give an overview of them all. In the first part we will discuss vitamin D analogs that are clinically approved (Table 2). For several conditions such as secondary hyperparathyroidism, psoriasis and osteoporosis, vitamin D analogs are frequently used as a treatment option. Paricalcitol **[2]** and doxercalciferol **[3]** are vitamin D_2_ analogs approved for therapeutic use of secondary hyperparathyroidism. In Japan falecalcitriol **[4]** and maxacalcitol **[5]** are also used to treat this disease. Secondary hyperparathyroidism is characterized by elevated parathyroid hormone (PTH) levels in response to hypocalcemia and is often caused by chronic kidney disease. Above-mentioned vitamin D analogs suppress PTH, as does 1,25(OH)_2_D_3_, but without inducing severe hypercalcemia. Clinical studies with chronic kidney disease patients show that different analogs induce a stronger PTH suppression compared to placebo treatment (Hamdy et al., 1995; Coburn et al., 2004; Coyne et al., 2006). Also, end-stage renal disease patients treated with these analogs often have a better survival (Teng et al., 2003; Tentori et al., 2006; Shinaberger et al., 2008). However, few studies with chronic kidney disease and end-stage renal disease patients directly compare the effects of 1,25(OH)_2_D_3_ with its analogs.

**Table 2:** **Overview of clinically approved vitamin D analogs**.

**Name**	**Structure**	**Indication**	**Brand name**
Tacalcitol (1α,24(R)(OH)_2_D_3_)	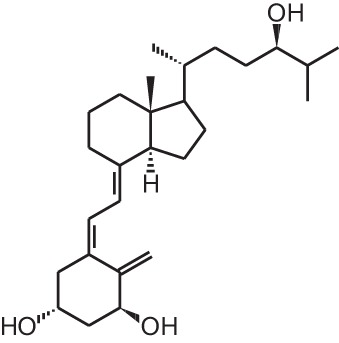	Psoriasis	Curatoderm® (Almirall Hermal), Bonalfa® (ISDIN, Teijin Pharma),…
Paricalcitol (19-nor-1α,25(OH)_2_D_2_)	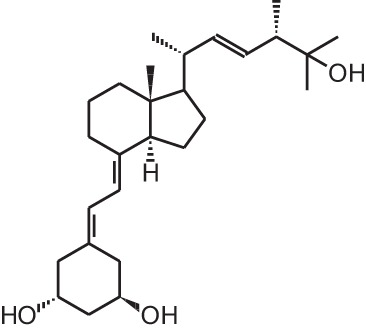	Secondary hyperparathyroidism	Zemplar® (Abbott)
Doxercalciferol (1α(OH)D_2_)	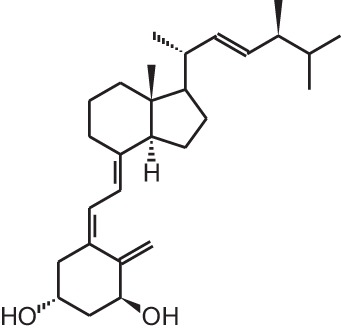	Secondary hyperparathyroidism	Hectorol® (Genzyme corp)
Falecalcitriol (26,27 F6-1α,25(OH)_2_D_3_)	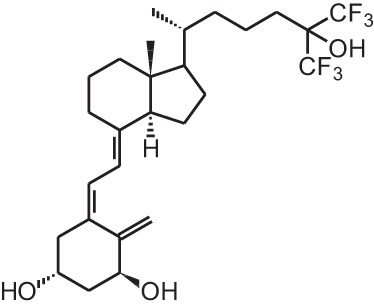	Secondary hyperparathyroidism (Japan only)	Fulstan® (Dainippon Sumitomo) and Hornel® (Taisho Yakuhin)
Maxacalcitol (22oxa-1α,25(OH)_2_D_3_)	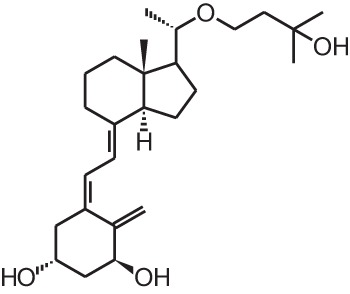	Secondary hyperparathyroidism and psoriasis (Japan only)	Oxarol® (Chugai Pharmaceutical)
Calcipotriol (22-ene-26,27-dehydro-1α,25(OH)_2_D_3_)	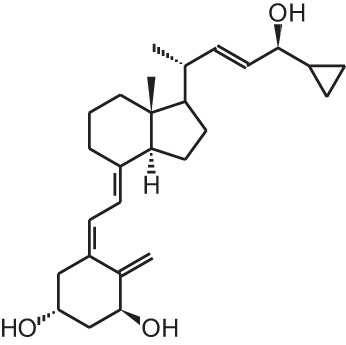	Psoriasis	Daivonex®, Dovonex® (LEO Pharma), Sorilux® (Stiefel)
Eldecalcitol (2β-(3-hydroxypropoxy)- 1α,25(OH)_2_D_3_)	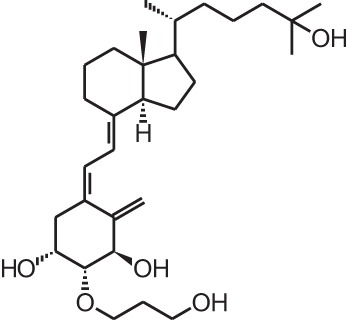	Osteoporosis (Japan only)	Edirol® (Chugai Pharmaceutical)

Psoriasis, a hyperproliferative condition of the skin, is also treated with vitamin D analogs. Tacalcitol **[6]**, calcipotriol **[7]** and the recently approved maxacalcitol **[5]** are used either as monotherapy or in combination with topical steroids such as betamethasone dipropionate to treat psoriasis. The analogs exert prodifferentiating and antiproliferative effects on keratinocytes and also possess important anti-inflammatory properties. Furthermore, alfacalcidol (**[**1α(OH)D_3_; **8]**, actually a pre-metabolite of 1,25(OH)_2_D_3_) and eldecalcitol (ED-71) **[9]** are used in Japan in the treatment of osteoporosis. The recently approved eldecalcitol **[9]** is more effective than 1,25(OH)_2_D_3_ and alfacalcidol **[8]** in increasing bone mineral density and mechanical strength in ovariectomized rats (Uchiyama et al., 2002). Various studies in mouse models as well as in patients show that treatment with eldecalcitol **[9]** leads to higher lumbar and hip bone mineral density and a lower incidence of new vertebral fractures (Ito et al., 2011; Matsumoto et al., 2011; Harada et al., 2012; Hagino et al., 2013), making eldecalcitol **[9]** a very promising new analog for the treatment of osteoporosis.

## Genome- and transcriptome-wide effects of vitamin D analogs

The exact mechanism of action of vitamin D analogs still has to be deciphered. The reason why specific analogs have superagonistic actions on specific tissues remains unknown, however several studies have tried to elucidate the mechanisms behind these tissue-specific effects. The catabolism of vitamin D analogs is one of the mechanisms that have an effect on their potency. Modifications of the side chain of 1,25(OH)_2_D_3_ are known to slow down its catabolism by CYP24A1 (Jones, 1997). Seocalcitol (EB1089) **[10]** and 20-epi-1,25(OH)_2_D_3_ **[11]** are degraded slower compared to 1,25(OH)_2_D_3_ leading to a longer exposure of these analogs to the tissues (Hansen and Maenpaa, 1997; Kissmeyer et al., 1997; Shankar et al., 1997; Zella et al., 2009). The metabolites formed after catabolism of lexicalcitol **[12]** are more active than the ones formed after 1,25(OH)_2_D_3_ is catabolized (Dilworth et al., 1997) and this analog is also more effective in slowing down the degradation rate of the VDR (van den Bemd et al., 1996). Moreover, since some cell types prefer specific catabolism pathways and enzymes above others, the degradation process may also contribute to the tissue-specific activity of vitamin D analogs. The affinity for the vitamin D binding protein (DBP) also plays a role in the activity of vitamin D analogs. Maxacalcitol **[5]** for example has a 500 times lower affinity for DBP and is thus cleared faster from the circulation than 1,25(OH)_2_D_3_ (Okano et al., 1989a). This analog has a short effect on bone and intestine, tissues responsible for calcium homeostasis, and a longer effect on PTH levels, making this analog ideal for the treatment of secondary hyperparathyroidism. However, it is still unknown why the duration of the effects is different in these tissues. Eldecalcitol **[9]** on the other hand has a higher DBP affinity compared to the mother compound, leading to longer sustained plasma levels and is thus more suitable for the treatment of osteoporosis (Okano et al., 1989b).

Another mechanism that contributes to the superagonistic effects of vitamin D analogs is their interaction with the VDR, co-activators and VDREs. 20-epi-1,25(OH)_2_D_3_, a C-20 epimer of 1,25(OH)_2_D_3_ **[11]**, promotes heterodimerization between VDR and retinoid X receptor (RXR) (Liu et al., 2001). 20-epi-1,25(OH)_2_D_3_ **[11]** and other analogs like maxacalcitol **[5]**, CD578 **[13]**, inecalcitol **[14]**, and TX527 **[15]** require lower concentrations to recruit specific coactivators to the VDR/RXR/VDRE complex (Liu et al., 2000; Eelen et al., 2005, 2008; Schwinn and DeLuca, 2007). Approximately 10- fold lower doses of inecalcitol **[14]** and TX527 **[15]** are needed, compared to 1,25(OH)_2_D_3_, to acquire the same amount of co-activator interactions (Eelen et al., 2005). Vitamin D analogs might also be able to induce tissue-specific effects by favoring binding to specific *VDRE* motifs in target gene promoters. Analogs with a 20-methyl group as well as seocalcitol **[10]** bound to a VDR/RXR complex preferably interact with the IP9 type of VDRE (Danielsson et al., 1996; Quack and Carlberg, 1999).

On the genome level, studies using chromatin immunoprecipitation (ChIP) and micro-array techniques have investigated 1,25(OH)_2_D_3_-regulated genes in different cell lines. One ChIP study compared the binding sites of the VDR in intestinal tissue after 1,25(OH)_2_D_3_ or 20-epi-1,25(OH)_2_D_3_ **[11]** treatment. This study shows that both 1,25(OH)_2_D_3_ and 20-epi-1,25(OH)_2_D_3_ **[11]** induce VDR binding to *CYP24A1* and *TRPV6* loci in the intestine, but the analog elicits a prolonged VDR binding to these genes leading to its superagonistic characteristics such as hypercalcemia *in vivo* (Zella et al., 2009). Other ChIP studies have tried to investigate the molecular mechanisms of some analogs in different tissues. In osteoblast cell models 2MD **[16]** bound to the VDR is able to bind VDREs at lower concentrations compared to 1,25(OH)_2_D_3_ (Yamamoto et al., 2003). Seocalcitol **[10]**, on the other hand, mediates the dissociation of Williams syndrome transcription factor of the aromatase promoter leading to inhibition of aromatase expression and activity in breast cancer cells which is one of the main therapeutic strategies in breast cancer patients (Lundqvist et al., 2013). In a recent paper binding sites of VDR and mothers against decapentaplegic homolog 3 (SMAD3) were investigated in hepatic stellar cells. These transforming growth factor β1 (TGFβ1)-activated cells play an important role in liver fibrosis. In this study it is shown that VDR and SMAD3 can at least transiently co-occupy the same genomic sites and function as enhancers of pro-fibrotic gene expression. However, when calcipotriol **[7]** is added, the TGFβ1-induced recruitment of SMAD3 is compromised and binding of VDR to these genomic sites is enhanced 10-fold meaning that liganded VDR antagonizes SMAD residency on chromatin and thereby suppresses pro-fibrotic gene expression (Ding et al., 2013). This genomic feedback circuit is a previously unknown mechanism of calcipotriol **[7]**.

Micro-array studies in various cancer cell lines such as leukemia, prostate, breast, colorectal, and ovarian cancer show that a variety of gene clusters are influenced by 1,25(OH)_2_D_3_ and its analogs (reviewed in Kriebitzsch et al., 2009). Cell growth, apoptosis, cellular adhesion and extracellular matrix composition, oxidative stress, immune function, intra- and intercellular signaling and steroid/lipid metabolism are frequently modulated processes in cells by 1,25(OH)_2_D_3_ and its analogs. However, when different cell lines are compared, few 1,25(OH)_2_D_3_/analog-regulated genes overlap, which suggests that 1,25(OH)_2_D_3_ and its analogs behave in a cell type- and tissue-specific way. Also studies using human T-cells (Baeke et al., 2011), rat ventricular heart tissue (Bae et al., 2011), and bone marrow-derived mouse dendritic cells (Griffin et al., 2004) have researched the impact of 1,25(OH)_2_D_3_ analogs on gene expression. In these studies genes important for cell growth, cell death and cell signaling are regulated, but also a large set of genes implicated in the migration of T-cells and dendritic cells are influenced. TX527 **[15]** imprints human T-cells with a migratory signature and targets them to sites of inflammation (Baeke et al., 2011). Paricalcitol **[2]** treatment of rats with cardiac hypertrophy prevents the progression of cardiac hypertrophy and the development into chronic heart failure. The genomic changes associated with cardiac hypertrophy in the ventricular heart tissue of these rats are, in part, reversed by paricalcitol **[2]** administration (Bae et al., 2011). Furthermore, other studies investigated if 1,25(OH)_2_D_3_ analogs are able to bind and regulate different genes compared to 1,25(OH)_2_D_3_. All conducted studies conclude that 1,25(OH)_2_D_3_ and its analogs induce or repress the same set of genes. Seocalcitol **[10]** induces a less malignant phenotype in SCC25 squamous cell carcinoma cells and modulates expression of genes important in cell cycle progression, cell adhesion, extracellular matrix composition, intra- and intercellular signaling, G-protein coupled function, redox balance, and steroid metabolism. In these cells, seocalcitol **[10]** regulates the same genes compared to 1,25(OH)_2_D_3_, however gene regulation by 1,25(OH)_2_D_3_ is more transient (Lin et al., 2002). Also WY1112, a seco-9,11-*bis*nor-17-methyl analog lacking the C-ring and with a 21-epi side chain which is fluorinated on C26 and C27 **[17]**, was investigated in MCF-7 breast cancer cells. Despite the 400-fold stronger antiproliferative capacity of WY1112 **[17]**, the same genes are upregulated after 1,25(OH)_2_D_3_ or WY1112 **[17]** treatment. However, the induction ability is much higher for the analog (Vanoirbeek et al., 2009). When treating human coronary artery smooth muscle cells with equal amounts of 1,25(OH)_2_D_3_ or paricalcitol **[2]** the same genes are regulated (Wu-Wong et al., 2007; Shalhoub et al., 2010). In conclusion, differences in action and capacity of vitamin D analogs are more due to their specific sensitivities to metabolism and their specific interaction with the VDR, co-activators and VDREs than from different gene regulations. However, to our knowledge no studies have yet looked into the potential differences elicited by analogs compared to 1,25(OH)_2_D_3_ in the fields of proteomics and epigenetics, which could help to understand the molecular mechanism of 1,25(OH)_2_D_3_ and its analogs on different cell and tissue types.

## Effects of vitamin D analogs in cancer

The use of 1,25(OH)_2_D_3_ for the treatment of cancer gained interest since many tissues express vitamin D metabolizing enzymes as well as the VDR and because 1,25(OH)_2_D_3_ has potent antiproliferative and prodifferentiating effects on normal and malignant cell lines. Several analogs evaluated *in vitro* show stronger antiproliferative and prodifferentiating effects compared to the mother compound in different cancer cell lines. These compounds are further evaluated in rodent models to assess their anti-cancer activity and safety *in vivo*. All *in vivo* studies using rodent cancer models that were published between 2007 and 2013 are summarized in Table 3. For studies preceding 2007, the reader is referred to other reviews (Eelen et al., 2007). In most studies the growth of the tumor is inhibited without inducing severe hypercalcemia when appropriate doses of vitamin D analogs are used (Abe et al., 1991; Kawa et al., 1996, 2005; Akhter et al., 1997, Blutt et al., 2000; Prudencio et al., 2001; Grostern et al., 2002; Flanagan et al., 2003; Albert et al., 2004a; Wietrzyk et al., 2004; Zhang et al., 2005; Fichera et al., 2007; van Ginkel et al., 2007; Ghous et al., 2008; Lee et al., 2008; Schwartz et al., 2008; Gonzalez-Pardo et al., 2010; Seubwai et al., 2010; Berkovich et al., 2013; Chiang et al., 2013). However, in some models the analog dose that is effective in inhibiting tumor growth also causes hypercalcemia and lower survival of the treated animals (Albert et al., 2004b). Not only tumor proliferation is modulated by vitamin D analogs, also apoptosis, angiogenesis, migration of tumor cells, etc. are affected by some analogs. In xenograft studies where apoptosis in the tumor was investigated after vitamin D analog treatment, apoptosis or the necrotic field in the tumor is augmented (James et al., 1998; VanWeelden et al., 1998; Hara et al., 2001; Vegesna et al., 2003; Lambert et al., 2010; Park et al., 2012). Inecalcitol (Hybrigenics, France) **[14]** treatment of mice with squamous cell carcinoma xenografts increases apoptosis in the tumors and this increase is higher for the analog compared to 1,25(OH)_2_D_3_, while the capacity of the analog to inhibit proliferation is equal compared to the mother compound (Ma et al., 2013). Most studies agree that vitamin D analogs also have an effect on tumor metastasis. Seocalcitol **[10]** reverses the growth-stimulatory effects of parathyroid hormone-related protein (PTHrP), which plays a major role in prostate cancer progression and metastasis, in a xenograft mouse model of prostate cancer. The same study shows that seocalcitol **[10]** also inhibits migration and invasion of these prostate cancer cells *in vitro* (Bhatia et al., 2009). This analog also reduces the number and surface area of bone metastasis originating from intracardially injected breast cancer cells (El Abdaimi et al., 2000). Vitamin D analogs are thus able to reduce the number and growth of metastasis originating from various types of cancer cells (Sato et al., 1984; Lokeshwar et al., 1999; Nakagawa et al., 2005; Park et al., 2012). However, in a study using mice with chemically induced breast cancer, the invasion capacity of the tumor after seocalcitol **[10]** treatment is not affected (Liska et al., 2012). The effect of vitamin D analogs on angiogenesis has also been studied *in vivo*, but here the results are more conflicting. Some studies show no effect of vitamin D analogs on angiogenesis (Oades et al., 2002; Valrance et al., 2007), while others find decreased angiogenesis of xenograft tumors. Intraperitoneal injections of inecalcitol **[14]** decrease the vascularity of xenografted prostate cancer cells (Okamoto et al., 2012) and oral treatment of colorectal tumors in rats with alfacalcidol **[8]** also inhibits tumor angiogenesis (Iseki et al., 1999). All *in vivo* studies conclude that vitamin D analogs inhibit tumor growth but vitamin D and its analogs often do not influence tumor number. Seocalcitol **[10]** was given as chemoprevention in a transgenic mouse model for androgen-independent prostate cancer. Tumor growth is inhibited, however, there is no prevention in the development of tumors (Perez-Stable et al., 2002).

**Table 3:** ***In vivo* studies in rodent cancer models treated with vitamin D analogs (intraperitoneal i.p.; subcutaneous s.c.) published between 2007 and 2013**.

**Cancer type**	**Dosage vitamin D analog**	**Duration of treatment**	**Outcome**	**References**
**Seocalcitol (22,24-diene-24,26,27-trishomo-1 α,25(OH)**_2_**D**_3_**)** 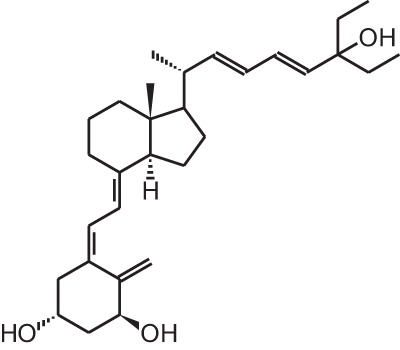				
Chemically-induced breast cancer	7 μg/kg/week	Approximately 80 days	Decreased tumor burden and volume	Liska et al., 2012
Chemically-induced breast cancer	Oral, 7 μg/kg/week	116 or 156 days	Prolonged latency of mammary gland tumors	Macejova et al., 2011
Prostate cancer xenograft	i.p., 0.5 μg/kg every other day	45 days	Reversal of growth stimulatory effects of PTHrP	Bhatia et al., 2009
Hepatocellular carcinoma xenograft	Oral and i.p., 0.02/0.1/0.5 μg/kg/d	Approximately 21 days	Inhibition of tumor growth	Ghous et al., 2008
Inoculation with mice breast cancer cells	i.p., 20 ng 3×/week	6 weeks	Inhibition of tumor growth, no inhibition of tumor angiogenesis	Valrance et al., 2007
**HY-11 (2-amino-3-deoxy-19-nor-22-ene-26-dihomo-27-dihomo-25(OH)D_3_**) 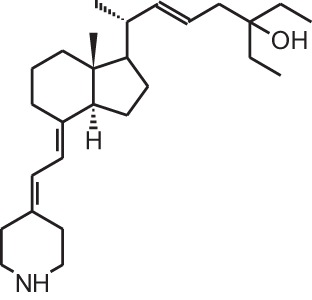				
Inoculation with mice leukemia cells	i.p., 10^−5^ M/d	26 days	50% increase in survival	Yoon et al., 2008
**Tacalcitol (1 α,24(R)(OH)**_2_**D**_3_**)** 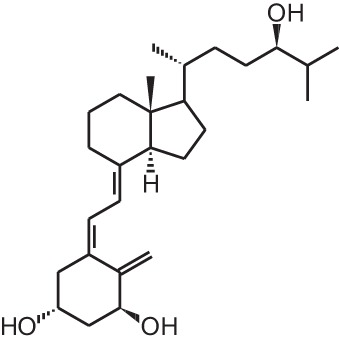				
Inoculation with mice colorectal cancer cells	Different concentrations s.c. (3 or 5×/week) or oral (3×/week) in combination with different concentrations of 5-fluorouracil	Variable duration	1 μg/kg/d optimal dose + prolongation of life span of mice (synergistic effect when combined with chemotherapy)	Milczarek et al., 2013a
Inoculation with mice or human colorectal cancer cells	s.c., Different concentrations in combination with different concentraties of irinotecan or oxaliplatin	Variable duration	Under certain experimental conditions vitamin D analogs and chemotherapy can work synergistically	Milczarek et al., 2013b
**Inecalcitol (19-nor-14-epi-23-yne-1 α,25(OH)**_2_**D**_3_**)** 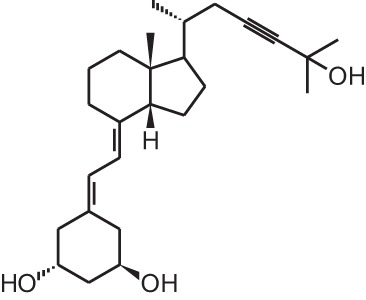				
Squamous cell carcinoma xenograft	i.p., 80/160/320 μg/mouse/d	3 days	Inhibition of tumor growth, increased apoptosis, decreased proliferation	Ma et al., 2013
Prostate cancer xenograft	i.p., 1300 μg/kg 3×/week	42 days	Delay of tumor growth, 50% decrease in tumor weight and decreased tumor vascularity	Okamoto et al., 2012
**TX527** (**19-nor-14,20-bisepi-23-yne-1 α,25(OH)**_2_**D**_3_****) 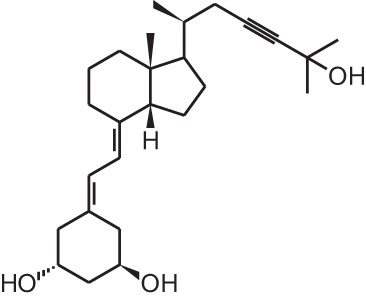				
Kaposi sarcoma xenograft	i.p., 10 μg/kg/d	4 days	Decreased tumor progression	Gonzalez-Pardo et al., 2010
**Paricalcitol (19-nor-1 α,25(OH)**_2_**D**_2_**)** 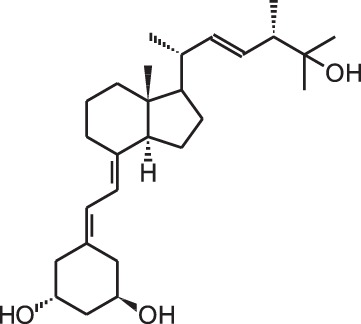				
Gastric cancer xenograft	s.c., 100 ng/d 3×/week	4 weeks	Lower tumor volume, reduced growth of intraperitoneal metastasis	Park et al., 2012
Pancreatic cancer xenograft	s.c., 2.5 μg/kg 3×/week	Variable duration	Inhibition of tumor growth	Schwartz et al., 2008
**Doxercalciferol (1 α (OH)D_2_**) 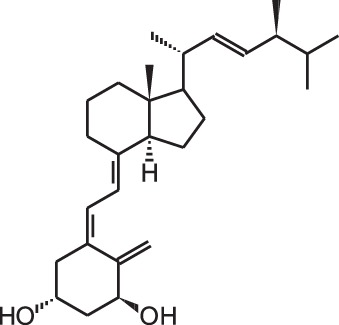				
Neuroblastoma xenograft	Oral, 0.15/0.3 μg/d	5 weeks	Lower tumor volume	van Ginkel et al., 2007
**Maxacalcitol (22oxa-1 α,25(OH)**_2_**D**_3_**)** 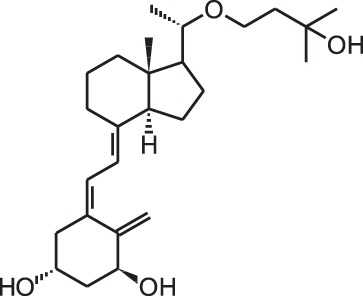				
Cholangiocarcinoma xenograft	i.p., 15 μg/kg/d	17 days	Inhibition of tumor growth	Seubwai et al., 2010
**Calcipotriol (22-ene-26,27-dehydro-1 α,25(OH)**_2_**D**_3_**)** 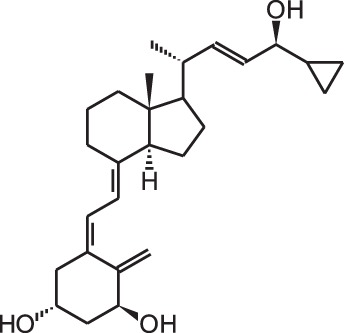				
UV-induced non-melanoma skin cancer	Topical application in combination with diclofenac and difluoromethylornithine	17 weeks	Decrease in number and area of tumors when combined with diclofenac	Pommergaard et al., 2013
**BGP-13 (1R, 3S, 5Z)-5-((8E)-2-((3R)-3-((2R, 3S)-3-(5-cyclopropyl-3H-1,2-dioxol-3-yl)-2-ethyl-3-methylcyclohexylidene) ethylidene)-4-methylenecyclohexane-1,3-diol)** 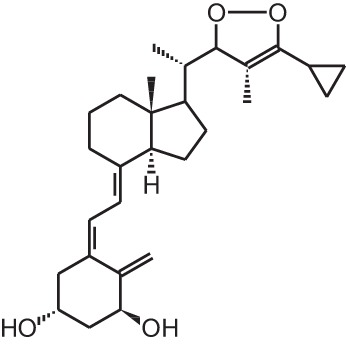				
Colorectal cancer xenograft	i.p., 2 μg/kg every 2 days	8 days	Inhibition of tumor growth	Berkovich et al., 2013
**PRI-2205 ((5E,7E)-22-ene-26,27-dehydro-1 α, 25(OH)**_2_**D**_3_**)** 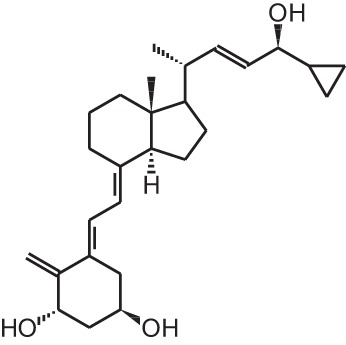				
Breast and lung cancer xenograft	s.c., 10 μg/kg 2 or 3×/week + cytostatics	18–21 days	Combination of analogs with low doses of cytostatics is not effective	Wietrzyk et al., 2007
**PRI-1906 ((24E)-(1S)-24-dehydro-24a-homo-1α,25(OH)**_2_**D**_3_**)** 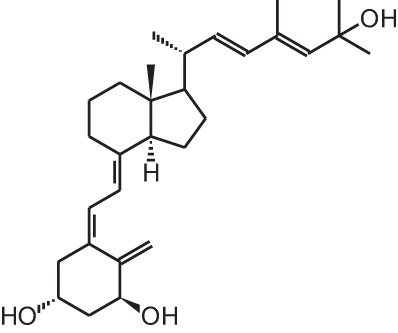				
Inoculation with mice breast cancer cells	s.c., 0.1 or 1 μg/kg/d	9 or 11 days	No effects	Wietrzyk et al., 2008
**BXL-01-0126 (20R-(4-hydroxy-4-methylpentyl)-23-yne-26,27-hexafluoro-19-nor-1 α, 25(OH)**_2_**D**_3_**)** 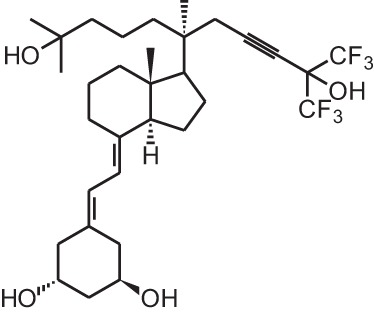				
Acute myeloid leukemia xenograft	i.p., 0.0625 μg	1 injection	Cathelicidin antimicrobial peptide present in systemic circulation	Okamoto et al., 2014
**BXL0124 (20R-21(3-hydroxy-3-deuteromethyl-4,4,4-trideuterobutyl)-23-yne-26,27-hexafluoro-1 α, 25(OH)**_2_**D**_3_**)** 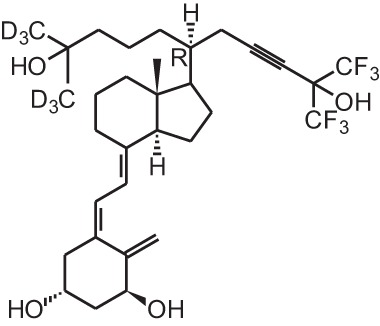				
Breast cancer xenograft	i.p., 0.1 μg/kg or oral 0.03/0.1 μg/kg 6 days/week	5 weeks	Suppressed tumor growth	So et al., 2011
Transgenic mice with breast cancer (ErbB2/Her-2/neu overexpressing tumors)	i.p., 0.3 μg/kg 3×/week	Approximately 38 weeks	Inhibition of tumor growth and regulation of ErbB2/AKT/ERK pathway	Lee et al., 2010
**Gemini0097 (20R-21(3-trideuteromethyl-3-hydroxy-4,4,4-trideuterobutyl)-23-yne-26,27-hexafluoro-19-nor-1 α, 25(OH)**_2_**D**_3_**)** 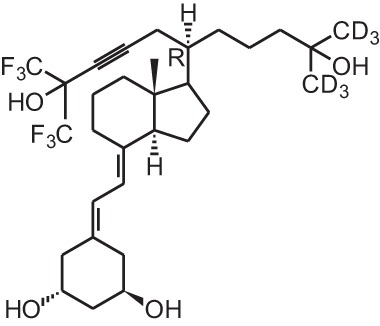				
ER-negative breast cancer xenograft	i.p., 0.1 μg/kg/d	9 weeks	Suppressed tumor growth	Lee et al., 2008
Chemically-induced breast cancer (ER positive)	i.p., 0.03/0.1/0.3 μg/kg 5days/week	9 weeks	Inhibition of tumor burden	
**MART-10 (19-nor-2 α-(3-hydroxypropyl)-1 α, 25(OH)**_2_**D**_3_**)** 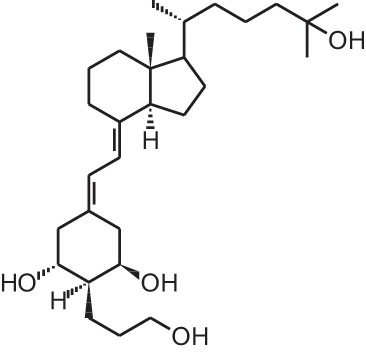				
Pancreatic cancer xenograft	i.p., 0.3 μg/kg 2×/week	3 weeks	Inhibition of tumor growth	Chiang et al., 2013
**1 α,25(OH)**_2_**D**_3_**-3-bromoacetate** 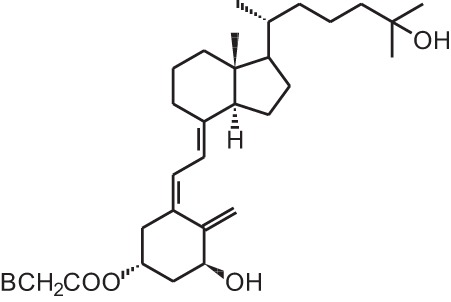				
Kidney cancer xenograft	i.p., 0.75 μg/kg every third day	80 days	Reduced tumor size and increased apoptosis	Lambert et al., 2010
**Ro26-2198 (16,23Z-diene-26,27-F6-19-nor-1 α, 25(OH)**_2_**D**_3_**)** 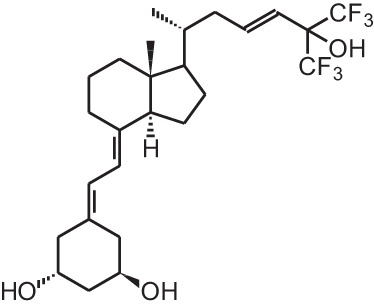				
Chemically-induced colorectal cancer	0.01 μg/kg/d via mini-osmotic pump	28 days	Inhibition of dysplasia progression and inhibition of proliferation and pro-inflammatory signals	Fichera et al., 2007

For studies preceding 2007 the reader is referred to other reviews (Eelen et al., 2007).

Since vitamin D and its analogs do not possess cytostatic properties, many *in vivo* studies have focused on vitamin D analog cancer treatment combined with radiotherapy and/or chemotherapy. When seocalcitol **[10]** is combined with radiotherapy in a xenograft model for breast cancer, the anti-cancer effects are more effective compared to monotherapy (Sundaram et al., 2003). Another analog, tacalcitol **[6]**, has been investigated in colorectal cancer xenograft in combination with different standard chemotherapies. Different concentrations as well as administration routes of tacalcitol **[6]** or PRI-2205 (an analog of calcipotriol) **[18]** were used in combination with different concentrations of 5-fluorouracil (5-FU). Using specific analog doses and chemotherapy schedules, a synergistic effect on the prolongation of the life span of the mice is achieved (Milczarek et al., 2013a). Also the combination with irinotecan or oxaliplatin was investigated. In this study the mice also show a longer life span and a stronger tumor growth inhibition compared to monotherapy when certain doses of tacalcitol **[6]** and chemotherapy were used. However, some combinations were more toxic than the monotherapies (Milczarek et al., 2013b). Other studies report better effects when combining calcipotriol **[7]** and diclofenac in a non-melanoma skin cancer model (Pommergaard et al., 2013). However, the combination of vitamin D analogs with chemotherapy does not always result in additive or synergistic effects. Combining maxacalcitol **[5]** and 5-FU did not enhance anti-tumor effects in a chemically induced breast cancer model (Iino et al., 1992). Another study investigated calcipotriol **[7]** and its derivatives in breast and lung cancer *in vivo* models and concluded that these analogs and low dose cytostatics are not effective in the used models (Wietrzyk et al., 2007). Also tacalcitol **[6]** in combination with cyclophosphamide does not lead to a significant difference in tumor growth inhibition compared to the vehicle treatment (Wietrzyk et al., 2008).

In view of the promising results that certain vitamin D analogs show against cancer *in vitro* and *in vivo* animal models, some analogs have been tested in cancer patients (Table 4). Seocalcitol **[10]** is an analog that has been extensively studied *in vitro* and *in vivo* in different cancer models, however in clinical trials the results are rather disappointing. Patients with advanced breast or colon cancer were treated with different doses of seocalcitol **[10]** (most patients tolerate 7 μg/d) but none of them showed a complete or partial response (Gulliford et al., 1998). Also oral seocalcitol **[10]** treatment in patients with inoperable pancreatic cancer exhibited no objective anti-tumor activity (Evans et al., 2002). Two out of 33 patients with inoperable hepatocellular carcinoma showed a complete response after oral seocalcitol **[10]** treatment, however the majority of the patients presented stable or progressive disease (Dalhoff et al., 2003). Inecalcitol **[14]** is in an early stage II of its clinical trial in chronic lymphocytic leukemia. Fifteen patients received 2 mg/d orally and one patient had a 90% decrease in blood lymphocyte count after 10 months of treatment, in 8 other patients blood lymphocyte count stopped growing when the treatment started (Hybrigenics, 2014). Intravenous administration of paricalcitol **[2]**, an analog that is approved for secondary hyperparathyroidism, also displayed no objective responses in patients with androgen-independent prostate cancer. However, elevated serum PTH levels, which are common for advanced prostate cancers, are reduced by the analog (Schwartz et al., 2005). Doxercalciferol **[3]**, also used in the treatment against secondary hyperparathyroidism, was investigated in androgen-independent prostate cancer patients. A phase I study administered oral doxercalciferol **[3]** between 5 and 15 μg/d, which was well tolerated by the patients (Liu et al., 2002). In the following phase II study, patients were treated with 12.5 μg/d for a minimum of 8 weeks and 30% of these patients experienced stable disease for over 6 months (Liu et al., 2003). Oral treatment of non-Hodgkin's lymphoma patients with 1 μg/d alfacalcidol **[8]**, a pre-metabolite of 1,25(OH)_2_D_3_, resulted in a low overall response. Out of 34 treated patients, only 4 had a complete response and 4 others showed a partial response to the treatment (Raina et al., 1991). Calcipotriol **[7]** is often used to treat skin psoriasis and has thus been investigated in patients with locally advanced or cutaneous metastases from breast cancer. In both studies the analog was applied topically at a dose of 100 μg/d. One study reported no response after 3 months of treatment (O'Brien et al., 1993), while in the other study 3 patients showed a 50% reduction in the diameter of treated lesions after 6 weeks (Bower et al., 1991). A more recently developed analog, ILX23-7553 **[19]**, was investigated in 16 patients with advanced solid tumors but no objective response was seen (Jain et al., 2011). Similar to the *in vivo* studies, clinical trials have also combined vitamin D analogs with standard radiotherapy or chemotherapy. Metastatic breast cancer patients were given oral paricalcitol **[2]** doses between 2 and 7 μg/d in combination with taxane-based chemotherapy and this regimen was well tolerated by the patients (Lawrence et al., 2013). Oral inecalcitol **[14]** was given to patients with hormone-refractory prostate cancer in combination with docetaxel for maximum 18 weeks. This study had a response rate of 85% based on a PSA decline of at least 30% within 3 months of treatment (Hybrigenics, 2014). In a small study with acute non-lymphoid leukemia patients the combination of alfacalcidol **[8]** and chemotherapy resulted in 17% of the patients with a complete response and 45% with a partial response (Petrini et al., 1991). The same analog was combined with standard treatment of surgery, radiotherapy, and/or chemotherapy in glioblastoma and anaplastic astrocytomas. Here, 0.04 μg/kg/d alfacalcidol **[8]** was administered resulting in 27% of the patients with progressive regression of the lesion and complete clinical remission (Trouillas et al., 2001). In metastatic renal cell carcinoma patients, oral treatment of 1 μg alfacalcidol/d **[8]** was combined with a 3 weekly administration of interferon-α for minimal 3 months. In these patients 25% had a partial response to the combination therapy (Obara et al., 2008). Randomized, placebo-controlled studies have been conducted with oral doxercalciferol **[3]** or alfacalcidol **[8]**. One study administered 10 μg/d doxercalciferol **[3]** or placebo during 4 weeks to patients with localized prostate cancer or high-grade prostatic intraepithelial neoplasia. However, no beneficial effects in serum or tissue markers were seen (Gee et al., 2013). Another study used the same dose in metastatic androgen-independent prostate cancer patients but combined the treatment with docetaxel. Also here, no enhanced PSA response rate or survival rate was seen after 4 weeks of treatment (Attia et al., 2008). Oral alfacalcidol **[8]** or placebo was given to patients with myelodysplastic syndromes. In the patients treated with the analog, a prolongation of leukemic transformation-free survival was seen compared to the placebo group (Motomura et al., 1991). Despite the promising *in vitro* and *in vivo* results of vitamin D analogs in cancer models, clinical trials have failed to proof the effects of vitamin D analogs in cancer patients. Vitamin D and its analogs lack cytotoxic activity, so using these analogs in combination with standard therapies such as radio- and chemotherapy is probably more effective than using the analogs as monotherapy. Next to the combination of analogs with standard cancer therapies, it is also possible that these analogs need to be given for a longer period of time or that treatment with analogs has to be started earlier, for example in early stages of disease or even as chemoprevention.

**Table 4:** **Clinical trials with vitamin D analog supplementation**.

**Cancer type**	**Sample size**	**Dosage vitamin D analog**	**Duration of treatment**	**Outcome**	**References**
**Seocalcitol (22,24-diene-24,26,27-trishomo-1 α, 25(OH)**_2_**D**_3_**)** 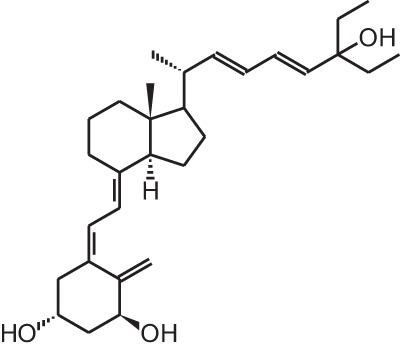					
Inoperable hepatocellular carcinoma	33	Oral individual dosage, most patients tolerate 10 μg/d	Up to 1 year	2 patients with complete response; 12 with stable disease; 19 with progressive disease	Dalhoff et al., 2003 (uncontrolled trial)
Inoperable pancreatic cancer	36	Oral individual dosage, most patients tolerate 10–15 μg/d	Minimum 8 weeks	No objective anti-tumor activity	Evans et al., 2002 (uncontrolled trial)
Advanced breast cancer and colorectal cancer	36	Individual dosage (solution), most patients tolerate 7 μg/d	From 5 days up to 1 year	No complete or partial responses	Gulliford et al., 1998 (uncontrolled trial)
**Inecalcitol (19-nor-14-epi-23-yne-1 α, 25(OH)**_2_**D**_3_**)** 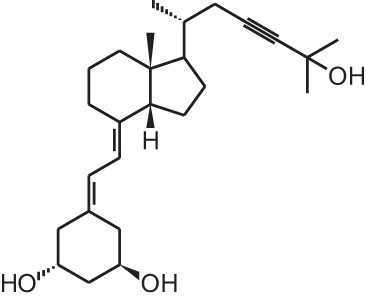					
Hormone-refractory prostate cancer	54	Oral individual dosage, maximum tolerated dose is 4 mg/d + docetaxel (chemotherapy)	Maximum 18 weeks	85% response rate based on a PSA decline of at least 30% within 3 months	Hybrigenics, 2014 (uncontrolled trial)
Chronic lymphocytic leukemia	15	Oral 2 mg/d	Not found	1 patient had a 90% decrease in blood lymphocyte count after 10 months of treatment; in 8 patients blood lymphocyte count stopped increasing when treatment was started; 6 patients showed no response	Hybrigenics, 2014 (uncontrolled trial)
**Paricalcitol (19-nor-1 α,25(OH)**_2_**D**_2_**)** 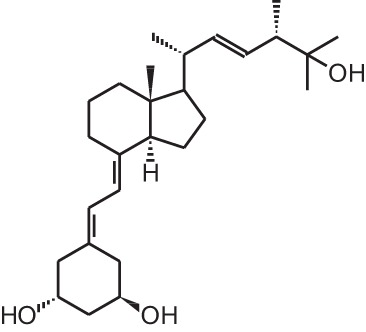					
Metastatic breast cancer	24	Oral individual dosage, 2–7 μg/d + taxane-based chemotherapy	8 weeks	Well tolerated regimen	Lawrence et al., 2013 (uncontrolled)
Androgen-independent prostate cancer	18	i.v., Individual dosage, 3×/week 5–25 μg	Up to 12 weeks	No objective response, reduced serum PTH levels	Schwartz et al., 2005 (uncontrolled)
**Doxercalciferol (1 α (OH)D**_2_**)** 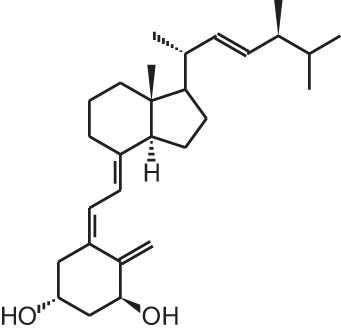					
Localized prostate cancer and high grade prostatic intraepithelial neoplasia	31	Oral, 10 μg/d	4 weeks	No beneficial effects in serum and tissue markers	Gee et al., 2013 (placebo-controlled)
Metastatic androgen-independent prostate cancer	70	Oral, 10 μg/d + docetaxel	4 weeks	No enhanced PSA response rate or survival	Attia et al., 2008 (placebo-controlled)
Advanced androgen-independent prostate cancer	26	Oral, 12.5 μg/d	Minimum 8 weeks	30% experienced stable disease for over 6 months	Liu et al., 2003 (uncontrolled)
Advanced androgen-independent prostate cancer	25	Oral individual dosage, 5–15 μg/d	Minimum 8 weeks	Well tolerated, maximal tolerated dose was not reached	Liu et al., 2002 (uncontrolled)
**Alfacalcidol (1 α (OH)D_3_**) 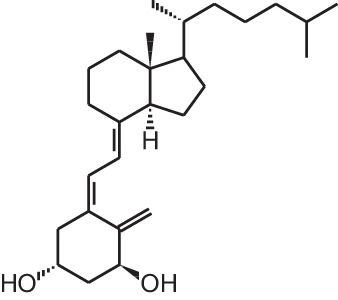					
Metastatic renal cell carcinoma	16	Oral, 1 μg/d + interferon-α (3×/week)	Minimum 3 months	25% had partial response	Obara et al., 2008 (uncontrolled)
Glioblastoma and anaplastic astrocytomas	11	0.04 μg/kg/d + surgery/chemotherapy/ radiotherapy	Not found	27% showed progressive regression of the lesion and had a complete clinical remission	Trouillas et al., 2001 (uncontrolled)
Myelodysplastic syndromes	30	Oral, 4–6 μg/d	Median 17 months	Prolongation of leukemic transformation-free survival	Motomura et al., 1991 (placebo-controlled)
Acute non-lymphoid leukemia	11	Analog + chemotherapy	Not found	17% complete remission, 45% partial remission	Petrini et al., 1991 (uncontrolled)
Progressive low-grade non-Hodgkin's lymphoma	34	Oral, 1 μg/d	Minimum 8 weeks	4 patients has a complete response, 4 other patients had a partial response	Raina et al., 1991 (uncontrolled)
**Calcipotriol (22-ene-26,27-dehydro-1 α, 25(OH)**_2_**D**_3_**)** 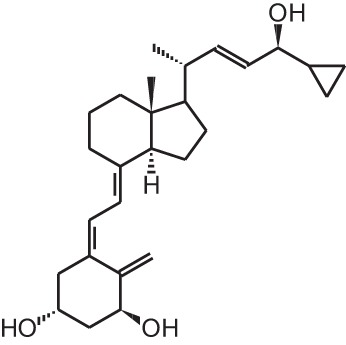					
Locally advanced or cutaneous metastatic breast cancer	19	Topical 100 μg/d	6 weeks	3 patients showed 50% reduction in diameter of treated lesions	Bower et al., 1991 (uncontrolled)
Locally advanced or cutaneous metastatic breast cancer	15	Topical 100 μg/d	3 months	No response	O'Brien et al., 1993 (uncontrolled)
**ILX23-7553 (16-ene-23-yne-1 α, 25(OH)**_2_**D**_3_**)** 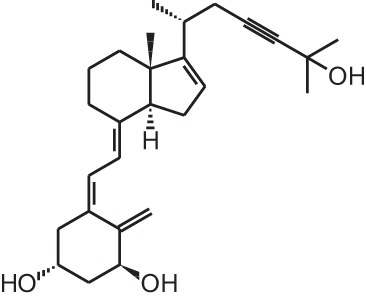					
Advanced solid tumors	16	Oral individual dosage, 1.7–37.3 μg/m^2^/d for 3 consecutive days, repeated in 7-day cycle	Minimum 3 weeks	No objective response	Jain et al., 2011 (uncontrolled)

## Conclusions and perspectives

Vitamin D and its analogs exhibit strong antiproliferative and prodifferentiating effects on different normal and malignant cell types. Several vitamin D analogs have been approved for treating psoriasis, osteoporosis, and secondary hyperparathyroidism and are often used as first or second-line treatment option. Despite promising *in vitro* as well as *in vivo* results in various cancer models, vitamin D analog treatment in clinical trials with cancer patients failed to prove efficacy in most trials. Different combinations of analogs and standard cancer therapies should be further explored as well as the correct duration and timing of administration. To unravel the exact working mechanisms of vitamin D analogs more research studies should compare the effects of vitamin D analogs in different cell types to the mother compound. Furthermore, differences between 1,25(OH)_2_D_3_ and its analogs are probably more due to their differences in metabolism and coactivator recruitment than from different genetic regulations. However, some fields such as epigenetics and proteomics remain largely unexplored in comparing the potentially distinctive effects of 1,25(OH)_2_D_3_ and its analogs. Since all current genomic and transcriptomic studies focus on established human cell lines, micro-array, and ChIP techniques comparing the effects of 1,25(OH)_2_D_3_ and its analogs on human primary tumor tissues should be investigated in the future.

### Conflict of interest statement

The authors declare that the research was conducted in the absence of any commercial or financial relationships that could be construed as a potential conflict of interest.
